# Sociodemographic and Clinical Variables of Depression among Patients with Epilepsy in a Neuropsychiatric Hospital in Nigeria

**DOI:** 10.1155/2020/2953074

**Published:** 2020-02-12

**Authors:** Nasir Olamide Madandola, Shehu Sale, Adebayo Sunday Adebisi, Ayodele Obembe, Auwal S. Salihu, Abdulfatai Tomori Bakare, Ishak Abioda Danjuma

**Affiliations:** ^1^Department of Psychiatry, Federal Neuropsychiatric Hospital, Kware, Sokoto, Nigeria; ^2^Department of Psychiatry, Usmanu Danfodiyo University Teaching Hospital, Sokoto, Nigeria; ^3^Department of Psychiatry, Aminu Kano Teaching Hospital, Kano, Nigeria

## Abstract

**Background:**

Depression is a major contributor to the global burden of disease. Its occurrence in patients living with epilepsy is not just common but also a serious comorbidity. Patients tend to suffer if the depressive disorder is undetected and thus untreated. The aim of this study is to estimate the prevalence of depressive disorder in patients with epilepsy. Also, the sociodemographic and clinical factors that are associated with the development of depression in people living with epilepsy were examined. *Materials and Method*. This was a descriptive cross-sectional study of participants living with epilepsy and receiving care at the Federal Neuropsychiatric Hospital, Sokoto, Nigeria. Participants were recruited consecutively as they come for follow-up care. A sociodemographic/clinical questionnaire and General Health Questionnaire version 28 (GHQ-28) were first administered to participants followed by the Composite International Diagnostic Interview (CIDI). The descriptive statistics were generated and analyzed. Logistic regression was also done to determine the predictors of depression in the study participants. All test of probability was set at *p* < 0.05.

**Results:**

A total of 400 participants with epilepsy were examined with GHQ-28 and CIDI. Out of the GHQ-28 examined individuals, 71 people (17.8%) met criteria for caseness while 35 participants (8.8%) were depressed when assessed with CIDI. The predictors of depressive illness in participants living with epilepsy were GHQ caseness (*p* < 0.05. *p* < 0.05. *p* < 0.05.

**Conclusion:**

Depression is common in people with epilepsy. Physicians should actively assess individuals with epilepsy for symptoms of depression. Special attention should be paid to patients with a family history of epilepsy and those from minority ethnic groups.

## 1. Introduction

Depression often complicates epilepsy as it is the most frequent psychiatric disorder associated with epilepsy [1]. The abnormal brain neuronal activity that causes epileptic seizures can also lead to depressive moods, and the stress of living with a chronic condition can worsen feelings of anxiety and depression [2–4]. Depressed patients are often not motivated and thus making effective management of epilepsy difficult [5]. Depression could be reactive to epilepsy where it is a form of negative feedback to the ongoing seizure disorder [6]. Sad feeling that is occasional is common and not strange to human beings but what separates it from a disease condition is the intensity, which is higher and for longer periods of time in depression and sometimes without any apparent reason [7–9]. Depression in epilepsy could also be before, during, or after seizure and it may not even have any relationship to the seizure.

However, depression in patients with epilepsy is underdiagnosed even among mental health professionals. The reasons for this include inadequate evaluation of and poor attention to the psychological states of patients with epilepsy by the treating physician. Furthermore, the management of patients with epilepsy by a large number of non-mental health professionals who do not have the expertise in eliciting these depressive symptoms portends a worse prognosis. The increasing prevalence of depression worldwide and the World Health Organization's (WHO) postulate of depression being the leading cause of death by 2020 makes this more worrisome among this subpopulation.

Evidence-based findings and knowledge of the prevalence of depression including the factors associated with its occurrence among patients with epilepsy will enhance the management of these patients by mental health professionals. This knowledge gap will also be reduced among non-mental health professionals that also treat epilepsy. It is believed that these findings will be influenced by the sociocultural background of the study setting and these are factors that will be useful to governmental/nongovernmental organizations in planning healthcare interventions and implementing policies for patients with epilepsy.

## 2. Aim

To determine sociodemographic and clinical factors associated with the occurrence of depression among patients with epilepsy in Sokoto, Northwest Nigeria.

### 2.1. Objectives

1. To determine the prevalence of depression among patients living with epilepsy and attending the Federal Neuropsychiatric Hospital, Kware, Sokoto State, Nigeria
2. To examine the sociodemographic and clinical factors that are associated with the occurrence of depression among patients with epilepsy in the Federal Neuropsychiatric Hospital, Kware, Sokoto State, Nigeria

## 3. Methodology

Ethical approval for this study was sought and obtained from the Ethical Committee of the Federal Neuropsychiatric Hospital, Kware, Sokoto, where the study was carried out. The sample size of 400 was derived using the sample size estimate for proportion [10, 11]. As the outpatient departments are combined for all neuropsychiatric disorders, those with diagnosed epilepsy for at least 6 months prior to their participation were recruited consecutively after seeking their informed consent. The diagnostic criterion used to define epilepsy was the International Classification of Diseases (ICD-10) codes for epilepsy and seizures. Participants were examined with the proforma questionnaire designed by the researchers using relevant literatures. Data on their sociodemographic characteristics and clinical variables such as frequency of seizure, age at illness onset, previous substance use disorder, presence of recurrent/uncontrolled seizure, current use of antiepileptic(s), previous hospital admission on account of seizure, fear of having seizure (assessed in three domains (no fear/rarely/always)), description of family social support (assessed in three domains (reduced/no change/increased)), family history of mental illness, etc. were obtained and analyzed. Participants were initially screened with General Health Questionnaire GHQ-28 (a self-administered questionnaire used to screen for the presence of mental illness). After administration of the GHQ-28, the participants were then administered the depression module of the Composite International Diagnostic Interview (CIDI) (a fully structured validated instrument for the assessment of psychiatric disorders). This was used to diagnose depressive disorder in the participants. It would have been difficult to reschedule these patients for another day for the administration of the CIDI. This is because quite a number of them came from afar, and they do not have access to mobile phone for ease of contact. The GHQ and CIDI were administered by one of the researchers who was trained by a CIDI certified trainer. The following inclusion criteria were observed for the study: (1) Participants were between 18 and 60 years. (2) Patients that were diagnosed with epilepsy as defined by ICD-10 codes for epilepsy and seizures. (3) All participants with clinical diagnosis of epilepsy were required to have done electroencephalography (EEG). They were recruited, whether their EEG results were positive or negative. (4) Patients that had been diagnosed and stable with epilepsy for at least 6 months.

### 3.1. Study Design

It was a descriptive, cross-sectional, hospital-based study.

### 3.2. Data Analysis

Data was analyzed using Statistical Package for Social Sciences (SPSS) version 20 software. Data were presented in tables to highlight the demographic, psychological, and clinical variables of concern, and their relationship with depression means and standard deviations (S.D) was used for descriptive statistics while the means of continuous data were compared using *t*-test. The chi-square (*χ*^2^) test was used to compare categorical variables, and logistic regression was used to determine the predictors of depression. All tests of significance were set at *p* < 0.05.

## 4. Results

### 4.1. Sociodemographic Characteristics of Participants with Depression

A total of 400 participants took part in the study of which 35 (8.8%) were diagnosed as having depression. Non-Hausas were significantly more likely to be depressed when compared to Hausas. The other sociodemographic findings are as shown in Table 1.

### 4.2. Psychological Characteristics of Participants in Both Groups (Depressed and Nondepressed)

Fear of having seizure, seizure worry, change in lifestyle, and family history of mental illness were associated with depression among the participants, see Table 2 for full details.

### 4.3. Clinical Characteristics of Participants in Both Groups (Depressed and Nondepressed)

Table 3 illustrates the clinical variables that were associated with depression in those with epilepsy. The presence of physical complications, lower mean duration since last seizure episode, and GHQ caseness were associated with depression.

### 4.4. Multivariate Logistic Regressions for the Predictors of Depression in the Study Groups

Logistic regression analysis was done for the association between the significant sociodemographic/clinical factors and depression. Only ethnicity, family history of mental illness, and GHQ caseness remained as predictors of depression, see Table 4.

## 5. Discussion

### 5.1. Sociodemographic Variables

The sociodemographic findings are similar to other studies done in some northern states of Nigeria [12]. The finding of the ethnic minorities being more likely to become depressed when compared to the major ethnic group is in keeping with an earlier study in Sokoto, Nigeria [13]. They reported that the major ethnic groups had less rate of depression when compared to the minority ethnic groups. Other studies had also buttressed this fact of ethnic minorities and depression proneness [14–16]. These studies concluded that ethnic minorities lack social support among some other disadvantages. The other reasons given were that ethnic minorities experience barriers to good treatment care, they have limited knowledge, and they underutilize treatment avenues.

There was also the possibility of ethnic minorities facing lots of stress as a result of their migration which may precipitate mental illness [17].

### 5.2. Depression among the Patients with Epilepsy

There has been much variability in the reported rate of occurrence of depression among seizure patients by different studies. This varied from 5% to as high as 70%. The 8.8% that was reported in this study is in keeping with the observations of studies that have a higher number of patients with controlled seizure (especially due to medications). They often report a prevalence that is equivalent to the ones seen in the general population [18, 19].

However, a population-based, case-control study found that depression was about 3.7 times more likely to occur before initial (first) seizure than subsequently [20]. A number of reasons may explain the pathophysiology and this dual-directional relationship between epilepsy and depression. Generally, abnormal activity of neurotransmitters especially serotonin, noradrenalin, dopamine, GABA, and glutamate and neuropathological changes in the frontotemporal regions with 5-HT1A reduction and dysfunction of the hypothalamic—pituitary—adrenal axis have been implicated in the occurrence of depression in epilepsy [21].

Depression is a leading cause of disability worldwide and is a major contributor to the overall global burden of disease [22]. This portends a poorer outcome and increased cost of treatment for epilepsy in individuals with untreated comorbid depression.

### 5.3. Factors Associated with Depression in the Patients with Epilepsy

Epileptic patients that were of the minority ethnic group and also those that had a family history of psychiatric illness were more likely to be depressed. The association with the ethnic minority is similar to the findings of a similar study, where increased rate of depression in ethnic minorities was found [23]. Another study which aimed among other things, to determine the demographic correlates of depression among patients with epilepsy reported that ethnic minority and being elderly were the predictors of having depression [24]. Individuals from a minority ethnic group may be subjected to discrimination and culture shock, especially in immigrant ethnic minorities. However, it has been reported that immigration alone does not cause serious problem but it becomes a very potent factor in mental illness when combined with other risk factors like unemployment, language difficulties, and the stress of migration [25]. All these may act as precipitants to the person that is already having a genetic loading evident by a positive family history. Majority of participants that had depression also had a family history of mental illness; this was similar to what was found in a similar study in Nigeria [26]. In another study, it was reported that a family history of depression was quite common among depressed patients with epilepsy [13]. It shows that there is an element of genetic association in those that are vulnerable to depression which may be aggravated by the epilepsy.

In a review of studies on psychiatric disorders and epilepsy, personal and/or family psychiatric history was reported to be associated with an increased risk of nonpsychiatric and psychiatric adverse effects to antiepileptic drugs (AEDs) [27]. Depression has been particularly associated with the use of barbiturates, vigabatrin, topiramate, and felbamate in patients being treated for epilepsy. The identification of patients who are more at risk of developing depression during AED therapy is therefore crucial in their management. A family history of depression is an important risk factor and should be ascertained before institution of treatment.

## 6. Conclusion

The rate of occurrence of depression in patients with epilepsy in this study is relatively high when compared to reported rates of other comorbid psychopathologies like anxiety disorders in people with seizure disorders. Mental health professionals should thus consider the regular assessment of depression in patients with epilepsy, especially those who have a family history of psychiatric disorder. This will enhance the early detection of possible comorbid depression among them. Also, patients with epilepsy of the ethnic minorities should be regularly assessed for sociodemographic factors that are negatively impacting their quality of life. Social interventions should be instituted for them when these stressors are identified.

## Figures and Tables

**Table 1:** Comparison of the sociodemographic characteristics of the depressed and nondepressed study groups (*n* = 400).

Variable	Depression group *n* (%) (*N* = 35)	No depression group *n* (*N* = 365)	*Χ* ^2^/*t*	D. F	*p* value
Age in years					
Mean (S.D)	30.37 (8.35)	28.60 (9.34)	*t* = −0.52	398	0.60
Gender					
Male	19 (7.1)	247			
Female	16 (11.9)	118	*Χ* ^2^ = 2.57	1	0.08
Occupation by					
Social class	13 (8.2)	146			
Unemployed/social class (others)	22 (9.1)	219	*Χ* ^2^ = 0.11	1	0.86
Marital status					
Married	15 (8.6)	159			
Others	20 (8.8)	206	*Χ* ^2^ = 0.01	1	0.94
Educational status					
Western	10 (6.6)	141			
Education/NIL	25 (10.0)	224	*Χ* ^2^ = 1.38	1	0.24
Ethnicity					
Hausa	26 (7.4)	327	*Χ* ^2^ = 7.21	1	0.01
Others	9 (10.0)	38			
Religion					
Islam	34 (8.6)	360			
Christianity	1 (16.7)	5	*Χ* ^2^ = 0.48	1	0.43

**Table 2:** Comparison of the psychological characteristics of the study participants in the depressed and nondepressed groups (*n* = 400).

Variables	Depression group *n* (%) (*N* = 35)	No depression *n* (*N* = 365)	*X* ^2^	D. F	*p* value
Fear of having seizure					
No	20 (57.1)	290			
Yes	15 (42.9)	75	9.12	1	<0.01
Seizure worry					
No	10 (28.6)	210			
Yes	25 (71.4)	155	10.84	1	<0.01
Change in lifestyle					
No	6 (17.1)	210			
Yes	29 (82.9)	155	20.98	1	<0.001
Social support					
Reduced	6 (17.1)	42			
No change	15 (42.9)	165	0.97	2	0.62
Increased	14 (40.0)	158			
Family history of mental illness					
No	20 (57.1)	292			
Yes	15 (42.9)	73	9.72	1	<0.01

**Table 3:** Comparison of the clinical characteristics of the study subjects in the depressed and nondepressed study groups (*n* = 400).

Variables	Depression group *n* (%) (*N* = 35)	No depression group *n* (*N* = 365)	*Χ* ^2^/*t*	D. F	*p* value
Seizure diagnosis					
Grand mal	25 (8.3)	277			
Others	10 (10.2)	88	*Χ* ^2^ = 0.34	1	0.56
Age at seizure onset (yrs)					
0–19	22 (17.6%)	240			
20–39	13 (15.8%)	115			
40–59	0	10			
≥50					
Range: 1–59					
Mean (S.D)	14.83 (8.50)	16.57 (10.70)	*t* = 0.93	398	0.35
Duration of seizure (yrs)					
0–19.9	24 (7.3%)	304			
20.0-39.9	9 (13.4%)	58			
40.0-59.9	24 (40%)	3			
Range: 0.17-42.50					
Mean (S.D)	15.22 (11.27)	11.94 (8.15)	*t* = −1.68	37.48	0.10
Duration since last seizure(days)					
0–100	30 (10.5%)	256			
≥101	5 (4.4%)	109			
Range: 1–5475					
Mean (S.D)	69.49 (155.20)	223.15 (573.41)	*t* = 2.41	45.62	0.02
Physical complications					
No	20 (6.3)	298			
Yes	15 (18.3)	67	*Χ* ^2^ = 11.76	1	<0.01
Seizure in the last one month					
No	12 (6.2)	183			
Yes	23 (11.2)	182	*Χ* ^2^ = 3.21	1	0.07
Psychoactive drug prior seizure					
No	32 (8.8)	333			
Yes	3 (8.6)	32	*Χ* ^2^ = 0.002	1	0.06
Current hard drug use					
No	34 (9.2)	337			
Yes	1 (3.4)	28	*Χ* ^2^ = 1.10	1	0.25
Seizure drug					
Monodrug	29 (8.0)	332			
Multiple drugs	6 (1.8)	33	*Χ* ^2^ = 2.38	1	0.13
Medical disorder					
No	30 (8.3)	330			
Yes	5 (12.5)	35	*Χ* ^2^ = 0.783	1	0.26
Previous hospitalization					
No	32 (9.0)	323			
Yes	3 (6.7)	42	*Χ* ^2^ = 0.276	1	0.43
Maternal pregnancy complications					
No	25 (8.8)	260			
Yes	1 (16.7)	5	*Χ* ^2^ = 0.504	2	0.78
Not reported	9 (8.3)	100			
Delivery-related complications					
No	24 (8.5)	258			
Yes	2 (22.2)	7	*Χ* ^2^ = 2.099	2	0.35
Not reported	9 (8.8)	100			
GHQ caseness					
No	6 (1.8)	323			
Yes	29 (40.8)	42	*Χ* ^2^ = 111.367	1	<0.001
EEG report					
Negative	17 (10.4)	146			
Positive	18 (7.6)	219	*Χ* ^2^ = 0.972	1	0.32

**Table 4:** Logistic regression analysis for the predictors of depression in the participants.

Predictor variable	*B*	*p* value	Exp(B)	Exp(B) (95% CI) lower	Exp(B) (95% CI) upper
Ethnicity	-1.63	**<0.01**	0.20	0.06	0.64
Fear of having seizure	-0.33	0.49	0.71	0.28	1.83
Worry about seizure	-0.27	0.60	0.76	0.29	2.05
Lifestyle change	-0.47	0.42	0.62	0.20	1.94
Family history of psychiatric illness	-1.10	**0.02**	0.33	0.13	0.84
Duration since last seizure	0.000	0.83	0.94	0.77	1.39
Seizure complication	0.67	0.17	1.98	0.77	5.09
GHQ caseness	-3.33	**<0.001**	0.04	0.01	0.11
Constant	1.847	0.015	6.112		

## Data Availability

The data used to support the findings of this study are available from the corresponding author upon request.

## Additional Points

*Limitations*. The use of self-reported questionnaires could lead to bias as most of the questions were about what had happened in the past. The participants might have forgotten the actual details of events, and due to the fact that cognitive decline is a common occurrence in epilepsy, the data collected from participants are prone to recall bias. Also, the study was a cross-sectional descriptive one and thus may not be sensitive to changes in the levels of the measurements over time. This study focused on patients in the hospital setting; therefore, the findings may not be representative of what obtains in the community.
